# Surgical Aortic Valve Replacement with Concomitant Aortic Surgery in Patients with Purely Bicuspid Aortic Valve and Associated Aortopathy

**DOI:** 10.3390/jcdd8020016

**Published:** 2021-02-10

**Authors:** Mevlüt Çelik, Edris A. F. Mahtab, Ad J. J. C. Bogers

**Affiliations:** Department of Cardiothoracic Surgery, Erasmus University Medical Center, 3015 GD Rotterdam, The Netherlands; m.celik@erasmusmc.nl (M.Ç.); a.j.j.c.bogers@erasmusmc.nl (A.J.J.C.B.)

**Keywords:** aortic valve replacement, surgical, aortic surgery, bicuspid aortic valve, Bentall

## Abstract

The bicuspid aortic valve (BAV) is the most common congenital cardiac malformation associated with aortopathy. The current study provides surgical clinical data on the patient characteristics and long-term survival of this less common adult purely BAV population undergoing surgical aortic valve replacement (SAVR) with concomitant aortic surgery. Adult patients with purely BAV who underwent SAVR and concomitant aortic surgery were included. Prevalence, predictors of survival, and outcomes for this patient population were analyzed. A total of 48 patients (mean age 58.7 ± 13.2 years, 33% female) with purely BAV underwent SAVR and concomitant aortic surgery between 1987 and 2016. The majority (62%) of the patients had pure aortic stenosis (AS). A total of 12 patients died. Survival was 92%, 73%, and 69% at 1, 5, and 20 years of follow-up. At 15 years of follow-up, the survival was close to that of the Dutch population, with a relative survival of 77%. Adult patients with a purely bicuspid aortic valve morphology undergoing SAVR and concomitant aortic root and/or ascending aorta present with excellent survival.

## 1. Introduction

Bicuspid aortic valve (BAV) disease is the most prevalent congenital heart defect, with approximately 1% prevalence in the general population [1,2]. A classification system for BAV from 304 surgical specimens showed an incidence of only 7% purely BAV in an autopsied population [3]. Although much data are present in patients with BAV and a raphe, data in patients with the less common purely bicuspid aortic valve are still relatively limited [3].

BAV is associated with aortopathies that lead to clinical manifestations such as aortic dilation, aneurysm, and dissection [4]. In the adult population, BAV patients undergoing aortic valve surgery are younger than the tricuspid aortic valve population. Patients with BAV present earlier with aortic stenosis (AS) and tend to undergo more frequent concomitant aortic surgery due to aortopathy [5].

Studies assessing the clinical profiles of BAV patients with a purely bicuspid aortic valve undergoing surgical aortic valve replacement (SAVR) and concomitant aortic surgery remain scarce. Therefore, the purpose of this study is to (i) describe the clinical characteristics of purely BAV patients undergoing SAVR with concomitant aortic surgery and (ii) assess the long-term survival and predictors of survival in this subpopulation of BAV patients.

## 2. Methods

### 2.1. Study Design

**Figure 1:**
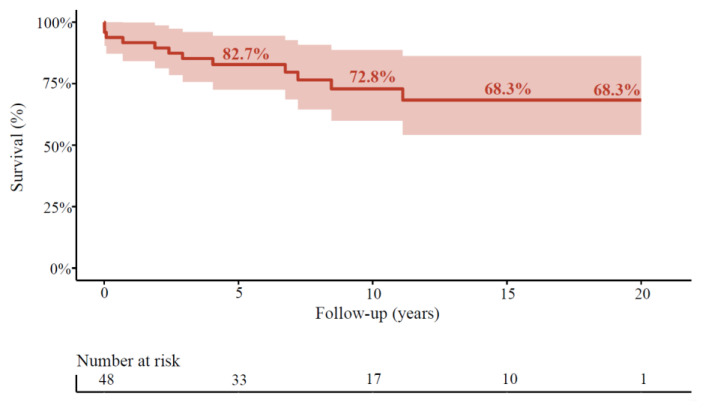
Long-term survival after surgical aortic valve replacement (SAVR) with aortic surgery. Survival in overall cohort. The shaded area represents the 95% confidence interval.

Patients older than 18 years of age undergoing SAVR between 1987 and 2016 at the Erasmus Medical Centre, Rotterdam, were included. In this SAVR population (total *n* = 4404), 16% of the patients who had undergone SAVR had purely BAV (*n* = 711), and only 7% of these purely BAV patients underwent concomitant aortic surgery (*n* = 48, as shown in Figure 1). Patients without pure BAV were excluded (Sievers 0) [3]. Likewise, patients without concomitant aortic surgery were excluded. Patients who did not receive a biological or mechanical aortic valve prosthesis were also excluded. The valvular morphology was classified during the operation and defined by the attending surgeon. Electronic medical records were used to obtain patient and procedural characteristics. For inclusion in this study, the bicuspid aortic valve was classified as a purely bicuspid aortic valve according to the Sievers classification (Sievers 0). All of the authors vouch for the validity of the data and adherence to the protocol.

### 2.2. Endpoints and Definitions

The primary aim was to assess the characteristics of patients with purely BAV requiring surgery. A further aim was to assess the survival after surgery of patients with purely BAV. The primary indication for operation (AS, aortic regurgitation (AR), or combined AS and AR) was determined based on the initial echocardiogram and according to the clinical guidelines in use at the time of the surgery.

### 2.3. Statistical Analysis

Discrete variables are presented as numbers, percentages, or proportions and compared with either the chi-square test or the Fisher exact test, where appropriate. Continuous variables are presented as means ± standard deviation or medians with the interquartile range (IQR) if there was evidence of skewed data according to the Kolmogorov–Smirnov test, and values were compared with either the two-sample *t*-test or Wilcoxon rank-sum test, where appropriate.

The relative survival can be used as an estimate of cause-specific mortality. It is defined as the ratio between the observed survival rates and the expected survival rates in the general population [6]. The Human Mortality Database was used to obtain the age-, sex-, and calendar year-matched expected survival data of the general population in The Netherlands [7]. The Human Mortality Database is continuously updated and includes mortality data from The Netherlands up until 2016. Relative survival was estimated through the Ederer II method [8,9]. Predictors of mortality were identified in a Cox proportional hazards model. Significant variables on univariable analyses were included in a multivariable Cox proportional hazards model. Sensitivity analysis was performed for isolated SAVR. Two-sided *p*-values < 0.05 were considered to be statistically significant. Data analyses were done using SPSS 25.0 (SPSS Inc, Chicago, IL, USA) and R software, version 3.5 (R Foundation, Vienna, Austria). Figures were generated using Microsoft Excel (Microsoft, Redmond, WA, USA) and R software, version 3.5 (R Foundation, Vienna, Austria).

## 3. Results

### 3.1. Characteristics of Patients with Bicuspid Aortic Valves

**Figure 2:**
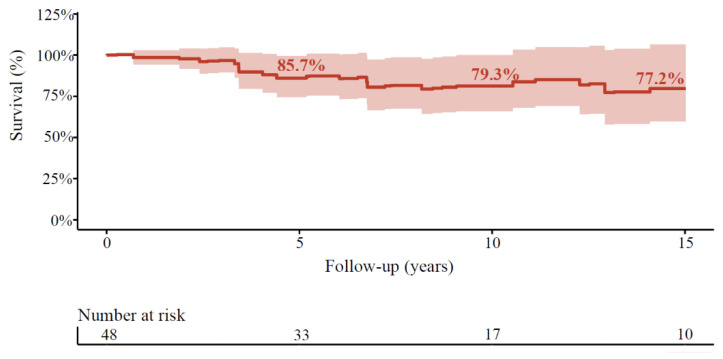
Long-term relative survival after SAVR with aortic surgery compared to the Dutch population. Relative survival compared to the age-, gender-, and year-matched population in the overall cohort. The shaded area represents the 95% confidence interval.

A total of 48 purely BAV patients underwent SAVR with concomitant aortic surgery (Figure 2). The mean age of operated patients was 58.7 ± 13.2, with 9 patients younger than 50 and 10 patients being 70 or older. The prevalence of comorbidities such as hypertension (32%), hypercholesterolemia (10%), and diabetes mellitus (4%) is shown in Table 1.

### 3.2. Procedural Characteristics

The indication for surgery was mainly AS (62%), followed by AR (19%) or combined AS and AR (19%). The type of aortic surgery was aortic root replacement in 21% of the patients and supracoronary ascendens replacement in 79% of the patients. Additionally, in 13 patients (27%), on top of the aortic root and/or ascending aorta, concomitant (hemi-)arch replacement was performed. Further concomitant surgery included coronary artery bypass graft (CABG) in 17% of the patients. The diameter of the implanted valve prosthesis was 24.8 ± 2.3. Further concomitant surgeries and characteristics are shown in Table 1.

### 3.3. Long-Term Outcomes after Surgery

**Figure 3:**
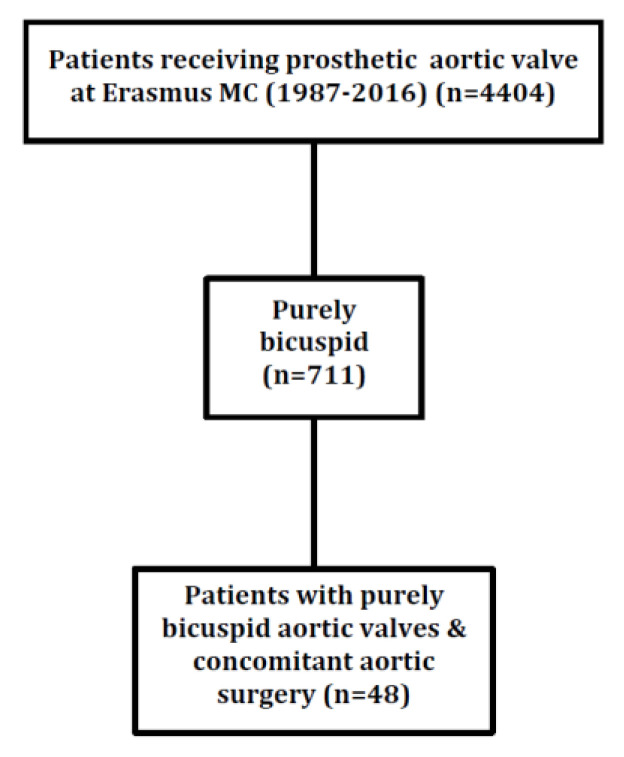
Flowchart of included patients.

A total of 12 patients died during follow-up. Survival was 92%, 90%, 83%, 73%, and 68% at 1, 2, 5, 10, and 20 years of follow-up in the overall cohort for patients with purely BAV (Figure 1). In age-, sex-, and year-matched Dutch controls, the relative survival in patients with purely BAV was 99%, 96% 86%, 79%, and 77%, at 1, 2, 5, 10, and 15 years of follow-up, respectively (Figure 3).

### 3.4. Factors Associated with Survival during Follow-Up in the Age-Matched Population

In univariable analyses, the presence of COPD was a predictor of survival (*p* = 0.02). However, cardiovascular risk factors such as increasing age (*p* = 0.20), atrial fibrillation (*p* = 0.80), and concomitant CABG (*p* = 0.64) were not predictors of survival (Table 2).

## 4. Discussion

This study describes patients’ characteristics and outcomes with purely bicuspid aortic valves that underwent aortic root, ascendens, and arch replacement as concomitant surgery to SAVR as the long-term survival and predictors of survival in this population. We found that the purely BAV population requiring SAVR and concomitant aortic surgery (i) mostly consists of young patients, (ii) has few cardiovascular risk factors that were not found predictive for their survival, and (iii) has excellent long-term survival.

The mechanisms leading to the development of BAV and the associated aortopathies are a matter of ongoing discussion. Adriana C. Gittenberger-de Groot and her team have performed an extensive number of indispensable studies on the spectrum of BAV disease and associated aortic anomalies over the past three decades. This contribution includes several developmental, histopathological, and anatomical studies on animal as well as human tissue, such as the meticulous explanation of cardiac development in congenital malformations [10], anatomical description of BAV and the aortic root [11], and the contribution of several cell lineages to the development of BAV and the associated aortic root anomalies [12,13,14]. In addition, other groups have reported that patient-specific factors such as aortic valve stenosis (AS), in combination with the specific leaflet morphology (the type of BAV) and the resultant shear stress, were associated with dilatation of the aorta [15,16].

Our cohort consisted of relatively young patients with purely BAV, a minority of the BAV population [3,17]. Our patients often presented with aortic stenosis, yet aortic regurgitation incidence was close to 40%. This prevalence of aortic regurgitation was higher than the standard surgical aortic valve replacement population, which could be partly due to aortic root or proximal aorta dilatation [4]. A bicuspid aortic valve indicates abnormal leaflet modeling, subsequently leading to turbulence downstream and upstream of the aortic valve [18]. This turbulence increases the aortic wall shear stress and abnormal helical flow in the ascending aorta, as shown with previous 4D magnetic resonance imaging [19,20]. In addition, increased matrix metalloproteinase activity in the aorta of BAV patients can affect the structural flexibility by altered elastin, collagen, and smooth muscle composition of the elastic laminae (aortic media) and can therefore lead to reduced compliance and increased aortic stiffness [4,21]. Therefore, BAV is associated with an increased prevalence of aortic root dilatation and ascending aortic aneurysms [22], even in patients without developed valvular dysfunction [23]. Moreover, gender differences in the aortic dimension of the patients with BAV were associated with aortopathy. Male patients more often present with larger aortic annulus and sinotubular junction dimensions [24]. The majority of our patients (two-thirds) described in this study were male. This finding is similar to previous studies [4]. Patients with bicuspid valves also present with fewer cardiovascular risk factors compared to patients with tricuspid aortic morphology and at an earlier age. This is partly due to accelerated calcification [25]. Aside from aortopathy and subsequent aortic dissection, endocarditis was prevalent in 4% of our cohort. This rate is lower than the previously noted higher prevalence [26].

In our cohort, the majority of the patients received mechanical valvular prostheses. Mechanical prostheses are undoubtedly superior regarding long-term durability and survival in the younger population [27]. However, a mechanical prosthesis might affect the patient’s quality of life due to anticoagulant medication and known bleeding risks, especially in an aging population [28,29]. Nevertheless, our cohort’s overall survival was exceptionally high, with 68% of the population surviving at 20 years of follow-up. The relative survival of this population (79%) is close to that of the Dutch general population. This could be partly explained by the lower prevalence in the cardiovascular risk profile of those patients.

### Limitations

Given its retrospective and nonrandomized nature, our study could be subject to shortcomings related to data capture and inherent confounders. Second, our study evaluated patient characteristics and long-term mortality as outcomes. Other aspects of clinical outcome and specific valve-related outcomes, including symptom improvement, quality of life, and structural valve dysfunction at long-term follow-up, were not assessed and should be assessed in further studies. In our study, we only included patients with purely BAV (Type 0) to create a homogenous BAV morphology. However, we are missing other BAV types with associated raphe and a comparison between our population and the TAV population in terms of the prevalence of aortopathy, clinical patient characteristics, and survival.

## 5. Conclusions

Patients with purely BAV undergoing SAVR with concomitant aortic surgery present with excellent survival rates. Additional studies are needed to examine the exact effect of the intervention on other endpoints, such as quality of life.

## Figures and Tables

**Table 1:** Baseline and procedural characteristics in the overall cohort.

	Overall Cohort
Age at operation	58.7 ± 13.2
Gender (female)	16 (33.3)
Indication	-
AS	30 (62)
AR	9 (19)
Combined	9 (19)
Previous cardiac operation	6 (13)
Creatinine	0.93 (0.86–1.07)
≥2 mg/dL	0
Atrial fibrillation	5 (10)
Diabetes mellitus	2 (4)
Decompensation cordis	4 (8)
Hypertension	15 (32)
Hypercholesterolemia	5 (10)
Previous myocardial infarction	3 (6)
Previous PCI	1 (2)
COPD	1 (2)
Endocarditis	2 (4)
History of cancer	3 (6)
Stroke/TIA	2 (4)
Stroke	1 (2)
TIA	2 (4)
Arterial disease	3 (6)
Carotid	1 (2)
Peripheral	2 (4)
Concomitant CABG	8 (17)
Aortic surgery	-
Aortic root replacement	10 (21)
Supracoronay ascendens replacement	38 (79)
Ascendens + Hemi(arch)	13 (27)
Valve size	24.8 ± 2.3
Urgency	-
Emergent	3 (7)
Not emergent	42 (93)
LVEF	-
Preserved	35 (76)
Mildly reduced	3 (7)
Moderately reduced	6 (13)
Severely reduced	2 (4)
Valve (mechanical)	29 (60)

AR, aortic regurgitation; AS, aortic stenosis; CABG, coronary artery bypass graft; COPD, chronic obstructive pulmonary disease; LVEF, left ventricular ejection fraction; PCI, percutaneous coronary intervention; TIA, transient ischemic attack.

**Table 2:** Predictors of survival after SAVR with concomitant aortic surgery.

Characteristics	Univariable HR (95% CI); *p*-Value
Age	1.04 (1.00–1.10); *p* = 0.20
Sex (female)	1.7 (0.5–5.4); *p* = 0.37
AS	1.7 (0.4–6.1); *p* = 0.44
AR	0.2 (0.01–3.3); *p* = 0.25
Hypertension	1.1 (0.3–3.7); *p* = 0.86
Hypercholesterolemia	1.0 (0.8–1.1); *p* = 0.29
Diabetes mellitus	1.4 (0.9–2.1); *p* = 0.14
Arterial disease	2.3 (0.3–18.4); *p* = 0.44
Renal failure	1.3 (0.6–3.0); *p* = 0.49
Previous MI	3.1 (0.7–14.3); *p* = 0.14
Previous PCI	1.1 (0.6–2.1); *p* = 0.74
Decompensated heart failure	0.8 (0.1–6.1); *p* = 0.82
LVEF < 50%	1.8 (0.5–6.2); *p* = 0.35
Atrial fibrillation	0.7 (0.1–6.0); *p* = 0.80
Previous stroke or TIA	2.0 (0.2–15.3); *p* = 0.52
COPD	1.9 (1.3–2.8); *p* = 0.002
Concomitant CABG	1.4 (0.4–5.1); *p* = 0.64
Emergent SAVR versus non-emergent	1.7 (0.2–13.5); *p* = 0.62
Mechanical prosthesis	1.0 (0.2–4.3); *p* = 0.97

AR, aortic regurgitation; AS, aortic stenosis; CABG, coronary artery bypass graft; COPD, chronic obstructive pulmonary disease; LVEF, left ventricular ejection fraction; MI, myocardial infarction; PCI, percutaneous coronary intervention; SAVR, surgical aortic valve replacement; TIA, transient ischemic attack.

## Data Availability

Data will not be made available.
